# Spatial genetic patterns and distribution dynamics of *Begonia grandis* (Begoniaceae), a widespread herbaceous species in China

**DOI:** 10.3389/fpls.2023.1178245

**Published:** 2023-05-10

**Authors:** Yan Xiao, Xing-Juan Li, Xiao-Long Jiang, Chun Li, Xiang-Peng Li, Wei-Ping Li, Dai-Ke Tian

**Affiliations:** ^1^ College of Life Sciences, Hunan Normal University, Changsha, China; ^2^ Shanghai Key Laboratory of Plant Functional Genomics and Resources, Shanghai Chenshan Botanical Garden, Shanghai, China; ^3^ College of Forestry, Central South University of Forestry and Technology, Changsha, China; ^4^ Vegetable Germplasm Innovation and Variety Improvement Key Laboratory of Sichuan Province, Horticulture Institute of Sichuan Academy of Agricultural Sciences, Chengdu, China; ^5^ Institute of Plant Conservation, Hunan Botanic Garden, Changsha, China

**Keywords:** phylogeography, species distribution modeling, glacial refugia, chloroplast DNA, population genetic structure, *Begonia*

## Abstract

**Introduction:**

*Begonia* L., one of the 10 largest plant genera, contains over 2,100 species, most of which have a very limited distribution range. Understanding the spatial genetic structure and distribution dynamics of a widespread species in this genus will contribute to clarifying the mechanism responsible for *Begonia* speciation.

**Methods:**

In this study, we used three chloroplast DNA markers (*ndh*F-*rpl*32, *atp*I-*atp*H, and *ndh*A intron), coupled with species distribution modeling (SDM), to investigate the population genetic structure and distribution dynamics of *Begonia grandis* Dryand., the species of *Begonia* with the widest distribution in China.

**Results:**

Thirty-five haplotypes from 44 populations clustered into two groups, and haplotype divergence began in the Pleistocene (1.75 Mya). High genetic diversity (*H*_d_ = 0.894, *H*_T_ = 0.910), strong genetic differentiation (*F*_ST_ = 0.835), and significant phylogeographical structure (*G*_ST_/*N*_ST_ = 0.848/0.917, *P* < 0.05) were observed. The distribution range of *B. grandis* migrated northwards after the last glacial maximum, but its core distribution area remained stable.

**Discussion:**

Combined, the observed spatial genetic patterns and SDM results identified the Yunnan-Guizhou Plateau, the Three Gorges region, and the Daba Mountains as potential refugia of *B. grandis*. BEAST-derived chronogram and haplotype network analysis do not support the Flora Reipublicae Popularis Sinicae and Flora of China for subspecies classification based on morphological characteristics. Our results support the hypothesis that population-level allopatric differentiation may be an important speciation process for the *Begonia* genus and a key contributor to its rich diversity.

## 1 Introduction

Climate oscillations in the Quaternary, in addition to other climatic factors such as the strengthening of the Asian monsoon, exerted a significant influence on species distribution and vegetation assembly (Hewitt, 2000; An et al., 2001; Hewitt, 2004). Southwest and subtropical China are among the most critical temperate biological relics of the Quaternary ice-age cycles. Given that subtropical (Central/East/South) China, including the hilly mid-elevation areas that lie between the Qinling Mountains-Huai River line (at c. 34°N) and the tropical South (at c. 22°N), was not covered by glaciers during the Quaternary, and because of its complex terrain, this region became a suitable habitat for plants and provided multiple refugia, such as the Yunnan-Guizhou Plateau, Hengduan Mountains, Nanling Mountains, Wuling Mountains, Qinling Mountains, and Daba Mountains. These refugia are also hotspots of plant diversity and endemism in today’s subtropical China (Qiu et al., 2011). The east-west oriented Nanling Mountains, Daba Mountains, and Qinling Mountains may serve as plant migration routes between China’s eastern and western regions, while the north-south trending Wuling Mountains, Luoxiao Mountains, and Wuyi Mountains facilitate the north-south migration of plants (Wang, 1992).

*Begonia* L., belonging to the family Begoniaceae, is widely dispersed in pantropical forest ecosystems across Africa, America, and Asia. With over 2,100 accepted species^1^ (Hughes et al., 2015, updated: December 6, 2022), *Begonia* is one of the ten largest plant genera and is also the genus with the fastest increase in the number of new species published in the last 20 years (Frodin, 2004; Moonlight et al., 2018; Tian et al., 2018). *Begonia* is one of the most diverse plant taxa, harboring a rich morphological diversity (Forrest and Hollingsworth, 2003; Tebbitt et al., 2006; Tian et al., 2018). One of the best-known flowers worldwide, *Begonia* has excellent ornamental value, particularly regarding foliage and flowers, and more than 15,000 cultivars have been raised to date (Tian et al., 2018). With few exceptions (e.g., *B. longifolia* Blume, *B. palmata* D.Don, *B. grandis* Dryand., *B. handelii* Irmsch., and *B. fimbristipula* Hance.), *Begonia* species have a restricted distribution range, especially these native to limestone karsts (Kiew, 2001; Tebbitt, 2005; Tebbitt et al., 2006; Hughes and Hollingsworth, 2008). The species of *Begonia* that have widespread distribution often have rich morphological diversity and atypical dispersal attributes, such as fleshy fruits or bulbils (Hughes and Hollingsworth, 2008). Additionally, given its rich diversity of morphological and habitat types, *Begonia* is highly valuable for conservation, speciation, and diversity research. Over recent years, *Begonia* has been employed as a model for understanding the evolution of species-rich genera (Thomas, 2010; Dewitte et al., 2011). The existing population genetic data indicate that *Begonia* has a strong population genetic structure, high genetic differentiation (*F*_ST_ range = 0.277–0.937), and limited gene flow (Matolweni et al., 2000; Hughes et al., 2003; Hughes and Hollingsworth, 2008; Nakamura et al., 2014; Twyford et al., 2014; Chan et al., 2018; Li et al., 2018; Tseng et al., 2019).

*Begonia grandis*, assigned to *Begonia* sect. *Diploclinium* (Lindl.) A.DC, is one of the few widely distributed begonias native to China. Its distribution ranges from c. 75 m to 3400 m elevation, 97.45° to 121.78° E longitude, and 22.98° to 40.67° N latitude (Li et al., 2014). It is the only true hardy and the most cold-resistant species of *Begonia* and can overwinter at temperatures as low as −27°C. *Begonia grandis* is a deciduous perennial with both underground tubers and aerial stems and is the only species of *Begonia* that produces asexual reproductive bulbils in the axils. *Begonia grandis* has a long history of cultivation in China owing to its high ornamental, medicinal, and cultural value (Li et al., 2014), and is also of considerable phylogenetics importance given its particular systematics placement. It is considered to be one of the basal groups of the clade “*Diploclinium* grade” (Rajbhandary et al., 2011; Thomas et al., 2011; Moonlight et al., 2018). *Begonia grandis* is classified into three subspecies and three varieties in Flora Reipublicae Popularis Sinicae (FRPS) (Ku, 1999). However, Flora of China (FOC) has only accepted three subspecies [*B*. *grandis* subsp. *grandis* Dryand., *B*. *grandis* subsp. *sinensis* (A. Candolle) Irmscher, and *B*. *grandis* subsp. *holostyla* Irmscher] (Gu et al., 2007). Although both morphological and molecular evidence (unpublished data) suggest that this species is markedly different from other begonias, the classification of its subspecies is still controversial, and its population history dynamics remain unknown.

In this study, we collected samples of *B. grandis* (including all three subspecies described in FOC) from a wide range of habitats and assessed its genetic variation and population structure based on three chloroplast DNA (cpDNA) markers (*ndh*F-*rpl*32, *atp*I-*atp*H, and *ndh*A intron), coupled with species distribution modeling (SDM). We aimed to (1) reveal the population genetic pattern of *B. grandis*, (2) infer its potential glacial refugia and dispersal corridor, and (3) provide a useful reference for subspecies classification, germplasm conservation, and utilization of this widespread species.

## 2 Materials and methods

### 2.1 Population sampling

A total of 352 individual *B. grandis* plants were sampled from 44 wild populations (8 per population) covering all native distribution regions in China except Tibet (**Table 1**). For each population, the collected samples were separated from each other by at least 10 m. The collected fresh leaf samples were immediately stored in silica gel for later use. The voucher specimens of each population were stored at the Herbarium of Shanghai Chenshan Botanical Garden (CSH).

**Table 1:** Sampling information, cpDNA genetic diversity, locality habitat suitability, and stability obtained from SDMs for *B. grandis*.

Pop Code	Samples Location	Lon (°E)	Lat (°N)	Voucher Number	Sub	cpDNA genetic diversity			SDM		
						*π* × 10^-3^	*H*_d_	Haplotypes (no. of individuals)	*N*_pre_	*N*_LGM_	*N*_stab_
AL	Anlong, Guizhou	105.65	25.19	LXJ120809_3	G	0	0	H1 (8)	0.57	0.59	0.97
CB	Chengbu, Hunan	110.11	26.32	XY130701_1	S	0	0	H7 (8)	0.56	0.4	0.84
DL	Dali, Yunnan	100.04	25.71	LXJ120823_1	G	0.88	0.43	**H26 (6)**, **H27 (2)**	0.38	0.2	0.82
DS	Dushan, Guizhou	107.7	25.94	LXJ120823_4	G	0	0	H7 (8)	0.63	0.56	0.93
EJX	Jingxing, Hebei	114.14	37.73	LXJ130619_1	G	0	0	**H14 (8)**	0.46	0.02	0.56
EM	Emeishan, Sichuan	103.39	29.56	TDK983	S	0	0	H1 (8)	0.57	0.46	0.89
ESZ	Suizhou, Hubei	113.01	31.48	DC130425_2	S	0	0	**H11 (8)**	0.53	0.46	0.93
EYX	Yuexi, Hubei	116.08	30.99	TDK1430	G	0	0	**H12 (8)**	0.42	0.23	0.81
FS	Fengshan, Guangxi	107.27	24.5	TDK1454	G	0	0	H7 (8)	0.34	0.63	0.71
GLS	Lushan, Jiangxi	115.96	29.55	XY130827_1	S	0	0	H1 (8)	0.31	0.25	0.94
HS	Hengshan, Hunan	112.68	27.3	XY130817_1	G	0	0	H16 (8)	0.36	0.44	0.92
JLS	Lingshan, Shanxi	111.96	36.88	XY130820_1	G	0	0	H16 (8)	0.21	0.02	0.81
JX	Jixian, Tianjin	117.56	40.19	XY130813_1	S	0.13	0.25	H3 (7), **H25 (1)**	0.43	0.01	0.57
JZ	Jiaozuo, Henan	113.36	35.43	HYB130524-1	G	0	0	H16 (8)	0.56	0.07	0.51
KM	Kunming, Yunnan	102.64	24.95	LXJ121019_1	G/S	1.02	0.93	H7 (1), **H28 (1)**, **H29 (2)**, **H30 (2)**, **H31 (1)**, **H32 (1)**	0.52	0.37	0.85
LA	Linan, Zhejiang	119.43	30.34	LXJ130721_1	G/S	0	0	H1 (8)	0.5	0.23	0.73
LB	Leibo, Sichuan	103.6	28.5	LXJ130719_1	S	0.22	0.43	H7 (2), H21 (6)	0.66	0.52	0.85
LC	Lichuan, Hubei	108.72	30.21	LXJ121016_1	S	0	0	H1 (8)	0.5	0.36	0.86
LG	Leigongshan, Guizhou	108.18	26.37	TDK1193	S	0	0	H7 (8)	0.59	0.38	0.79
LJ	Lijiang, Yunnan	100.18	28.81	TDK1167	S	0.44	0.43	H22 (6), **H33 (2)**	0.09	0.02	0.93
LP	Liping, Guizhou	109.17	26.33	LXP130822_1	G	0	0	**H9 (8)**	0.62	0.56	0.94
LY	Lingyuan, Liaoning	119.17	40.66	SYG130526_1	G	0.26	0.25	H3 (7), **H20 (1)**	0.3	0.02	0.72
ML	Muli, Sichuan	101.18	28.05	LXJ120623_1	G	0.88	0.43	H22 (6), **H23 (2)**	0.41	0.15	0.74
MLP	Malipo, Yunnan	104.81	23.14	LXJ131007_1	G	0.13	0.25	H7 (7), H21 (1)	0.37	0.29	0.93
SNJ	Shennongjia, Hubei	110.69	31.82	TDK494	G	1.37	0.54	H7 (3), **H10 (5)**	0.61	0.36	0.75
SY	Shangyou, Jiangxi	114.18	26.01	TDK519	G	0	0	**H19 (8)**	0.52	0.34	0.82
SYX	Yixing, Jiangsu	119.67	31.3	LXJ130715_1	G	0	0	**H18 (8)**	0.46	0.3	0.84
TN	Taining, Fujian	117.19	26.97	TDK660	G	0.26	0.25	**H4 (7)**, H5 (1)	0.43	0.29	0.85
TS	Taishan, Shandong	117.08	36.31	LXJ130616_1	G	0	0	**H24 (8)**	0.53	0.09	0.56
TZ	Tongzi, Guizhou	106.87	28.28	TDK1117	S	0	0	H7 (8)	0.6	0.55	0.95
WX	Wenxian, Gansu	105.27	32.7	LXJ120928_4	G	0.64	0.25	H7 (7), **H8 (1)**	0.67	0.62	0.95
WYS	Wuyishan, Fujian	117.94	27.65	LXJ120924_1	G	0	0	H1 (8)	0.43	0.29	0.86
WZ	Wenzhou, Zhejiang	121.09	28.36	LXJ120921_2	H	0	0	**H35 (8)**	0.33	0.32	0.99
XC	Xichuan, Henan	111.05	33.27	LXJ120926_4	G	0	0	H3 (8)	0.6	0.5	0.9
XL	Xinglong, Hebei	114.14	37.73	LXP130824_1	S	0.64	0.25	H3 (1), **H15 (7)**	0.46	0.02	0.56
XLS	Longshan, Hunan	109.3	29.16	LXP130821_1	S	0	0	H7 (8)	0.65	0.59	0.93
XN	Xiuning, Anhui	118.04	29.81	TDK1172	S	0	0	H1 (8)	0.47	0.36	0.89
XS	Xiangshan, Beijing	116.18	39.99	LXJ120911_7	H	0.27	0.54	**H2 (3)**, H3 (5)	0.68	0.05	0.36
XSZ	Sangzhi, Hunan	109.81	29.68	TDK1506	S	0	0	**H17 (8)**	0.52	0.36	0.84
YA	Yaan, Sichuan	102.78	30.05	LXJ120916_3	H	0	0	H1 (8)	0.58	0.59	0.98
YS	Yangshan, Guangdong	112.46	24.41	TDK1371	S	0	0	**H6 (8)**	0.53	0.4	0.87
YT	Yongtai, Fujian	119.1	25.89	LXJ120918_1	H	0	0	H1 (8)	0.42	0.47	0.95
ZD	Zhongdian, Yunnan	100.03	27.5	LXJ120809_1	G	0.13	0.25	H7 (7), **H34 (1)**	0.35	0.11	0.77
ZG	Zigui, Hubei	110.93	30.75	GYF130521_1	G	3.56	0.61	H5 (5), H7 (1), **H13 (2)**	0.64	0.5	0.86

Pop code, population code; Sub, subspecies identification results based on morphological classification (G: *B. grandis* subsp. *grandis*, S: *B. grandis* subsp. *sinensis*, H: *B. grandis* subsp. *holostyla*); *H*_d_, haplotype diversity; π, nucleotide diversity; *N*_Pre_, present habitat suitability; *N*_LGM_, LGM habitat suitability; *N*_stab_, habitat stability since the LGM; Unique haplotypes are shown in bold.

### 2.2 DNA extraction, PCR amplification, and sequencing

Total genomic DNA was extracted using the DNAsecure Plant Kit (Tiangen Biotech, Beijing, China), following the manufacturer’s protocol. In a preliminary investigation of chloroplast variation, 24 cpDNA markers were sequenced for 12 samples from 6 natural localities. Because the *ndh*F-*rpl*32 (Thomas, 2010), *atp*I-*atp*H, and *ndh*A intron (Shaw et al., 2007) chloroplast regions were found to display greater variation than the other markers examined, these regions were subsequently amplified for all individuals using the same primer pairs (**Supplementary Table S1**).

PCR amplification was carried out in 20-μL volumes containing 11 μL of ddH_2_O, 4 μL of 5× Fast HiFidelity PCR buffer, 2 μL of DNA template, 1 μL of 20× Fast PCR Enhancer, 0.8 μL of each forward and reverse primer, and 0.4 μL of Fast HiFidelity Polymerase (Tiangen Biotech). PCR was carried out using the following program: An initial denaturation at 94°C for 2 min, followed by 35 cycles of denaturation at 94°C for 15 s, annealing at 60°C for 10 s, and elongation at 68°C for 30 s, with a final elongation step at 68°C for 5 min. The amplification products were sequenced by Shanghai Maipu Biotechnology Co., Ltd. and Sangon Biotech Co., Ltd. (both in Shanghai, China).

Nucleotide sequences were manually edited in SeqMan (Swindell and Plasterer, 1997), and then aligned using MEGA5 (Tamura et al., 2011) with subsequent manual adjustment with BioEdit v7.0.4.1 (Hall, 1999).

### 2.3 Genetic diversity and phylogeographic structure analysis

The cpDNA haplotypes, polymorphic sites, haplotype diversity (*H*_d_), and nucleotide diversity (*π*) were calculated using DnaSP v5.10 (Librado and Rozas, 2009). Haplotype spatial distribution maps were generated using ArcGIS 10.5.^2^ Permutation tests, implemented in PERMUT v1.0 (Pons and Petit, 1996), were employed to calculate total gene diversity (*H*_T_), within-population gene diversity (*H*_S_), *G*_ST,_ and *N*_ST_ based on 1,000 random permutations. *G*_ST_ is an unordered measure of genetic differentiation based solely on haplotype frequencies that do not incorporate phylogenetic distance, while *N*_ST_ estimates genetic differentiation based on considering similarities among different haplotypes that incorporate phylogenetic distance. A value for *G*_ST_ significantly lower than that for *N*_ST_ indicates the presence of phylogeographic structure. Analysis of molecular variance (AMOVA) was computed in Arlequin 3.5 (Excoffier and Lischer, 2010). A median-joining network was constructed using Network 5.0.1.1 (Bandelt et al., 1999) to evaluate phylogenetic relationships among haplotypes. Bayesian Analysis of Population Structure (BAPS) (Corander et al., 2008), a spatial clustering model, was employed to detect clusters of genetically similar populations and the spatial clustering of DNA sequences. Possible historical demographic expansions were examined under neutrality tests using Tajima’s (1989) *D* and Fu's (1997) *Fs* statistics. *D*-values significantly different from 0 are usually correlated with selection, bottlenecks, or population expansion, while a significantly negative Fs value indicates a recent demographic expansion. Mismatch distribution analysis (Schneider and Excoffier, 1999) was also used to infer the demographic histories of the species. Unimodal pairwise mismatch distributions indicate that populations have experienced recent demographic expansion, while multimodal distributions are related to demographic equilibrium or decline (Slatkin and Hudson, 1991; Rogers and Harpending, 1992). The raggedness index (*H*_Rag_) and *P*-values were computed to test the significance of the population expansion model. All these analyses were conducted in Arlequin 3.5 (Excoffier and Lischer, 2010). The estimates of pairwise genetic distance (*F*_ST_) were regressed against geographic distance using a Mantel test with 999 random permutations in GenAlEx 6.5 (Peakall and Smouse, 2012) to test for isolation by distance (IBD) patterns among populations.

### 2.4 Phylogenetic analysis and estimation of divergence times

Phylogenetic relationships of the haplotypes and the outgroup (*B. pedatifida* H. Léveillé) were reconstructed using a Bayesian approach implemented in BEAST v1.8.2 (Drummond et al., 2012). The divergence time was estimated using the GTR + I substitution model based on the Akaike information criterion (AIC) selected by IQ-TREE ModelFinder (Kalyaanamoorthy et al., 2017). As there is no fossil record or information regarding specific substitution rates for *Begonia*, a mean of 2 × 10^−9^ substitutions/site/year (s/s/y) was set for cpDNA based on the range of the synonymous substitution rates of chloroplast genes (1 × 10^−9^ to 3 × 10^−9^ s/s/y) (Wolfe et al., 1987) to estimate divergence time. An initial MCMC chain was run for 1 × 10^7^ generations to select optimal parameters for the BEAST analysis. Tracer v1.5^3^ (Rambaut and Drummond, 2007) was used to examine the parameter ucld.stdev. A value greater than 0 for this parameter suggests that the data were appropriate for an uncorrelated lognormal relaxed-clock model. All effective sample sizes of each parameter from Tracer v1.5 were ensured to be greater than 200. The final MCMC chain was run for 1 × 10^7^ generations with sampling every 1,000 generations using an uncorrelated lognormal relaxed-clock model and a constant population size. The maximum clade credibility (MCC) tree was generated with a 10% burn-in using TreeAnnotator v1.6.1 (Drummond and Rambaut, 2007). Finally, these results were compiled into a single tree visualized in Figtree v1.3.1.^4^

### 2.5 Species distribution model and visualizing dispersal corridors

Under the assumption that the climatic preference of *B*. *grandis* has not changed since the last glacial/interglacial cycle and will not change in the future (Nogués-Bravo, 2009), maximum entropy modeling performed in MaxEnt v3.4.1 (Phillips et al., 2006) was used to compare the geographic distribution of *B. grandis* in the present with that in the last glacial maximum (LGM) and the future. All herbarium specimens were validated before being used for data analysis. After removing duplicate records, 332 points of *B. grandis* were obtained from field collections, the Chinese Virtual Herbarium,^5^ and the National Specimen Information Infrastructure of China.^6^

Nineteen bioclimatic variables at a 2.5-arcmin resolution for the present (1960–1990), the LGM (approximately 22,000 years ago, Community Climate System Model [CCSM]), and the future (2060–2080, RCP8.5) were downloaded from WorldClim 1.4.^7^ Multicollinearity among variables was measured using a Pearson correlation matrix estimated in R v3.6.0.^8^ Nine climate variables were obtained when subsets of variables with high correlations (*r* > 0.8) were reduced to single variables (**Supplementary Table S2**). Potential species distributions were calculated using the mean of 10 duplicate results with random seeds. The area under the curve (AUC) calculated from a receiver operating characteristic (ROC) was used to evaluate the model performance. The AUC ranged between 0.5 and 1, with 0.5 representing a random prediction and 1 representing the maximum projection. Species distribution maps for the present and other periods were created based on the maximum training sensitivity plus specificity threshold (Jiménez-Valverde and Lobo, 2007). Three indices were calculated to compare changes in species distributions, namely, the habitat distribution area ratio (*N*_a_), habitat expansion extent (*N*_e_), and locality habitat stability (*N*_Stab_). *N*_a_ = (distribution areas of the present)/(distribution areas of the LGM or future), where a value close to 1 denotes a stable distribution, and a value noticeably higher or lower than 1 denotes a change in the distribution region of a species. *N*_e_ = [1 − (overlap distribution areas of the LGM or future and present/present distributions area)] × 100% represents the percentage of expansion or contraction between the LGM or future and the present. *N*_Stab_ = 1 − | *N*_Pre_ − *N*_LGM_|, where *N*_Pre_ and *N*_LGM_ mean habitat suitability of the present and LGM distribution area, respectively.

SDMtoolbox (Brown, 2014) in ArcGIS 10.5 was used to map the dispersal routes of *B. grandis* since the late Quaternary. The specific steps for this method were as follows: Firstly, a resistance layer was created by inverting the SDMs (1−SDM), and the resistance layer was then used to create a cost distance raster for each sample locality. Secondly, corridor layers were created between two localities that only shared haplotypes using the cost distance raster. Thirdly, the least cost path (LCP) method was used to better depict environmental heterogeneity in dispersal. The value of each corridor layer was classified as low, medium, or high, and then these three levels were subdivided into new values (5, 2, and 1). Finally, all of the reclassified corridor layers were summarized and standardized from 0 to 1, and the dispersal corridors of *B. grandis* were eventually identified.

## 3 Results

### 3.1 Genetic diversity and structure

**Figure 1:**
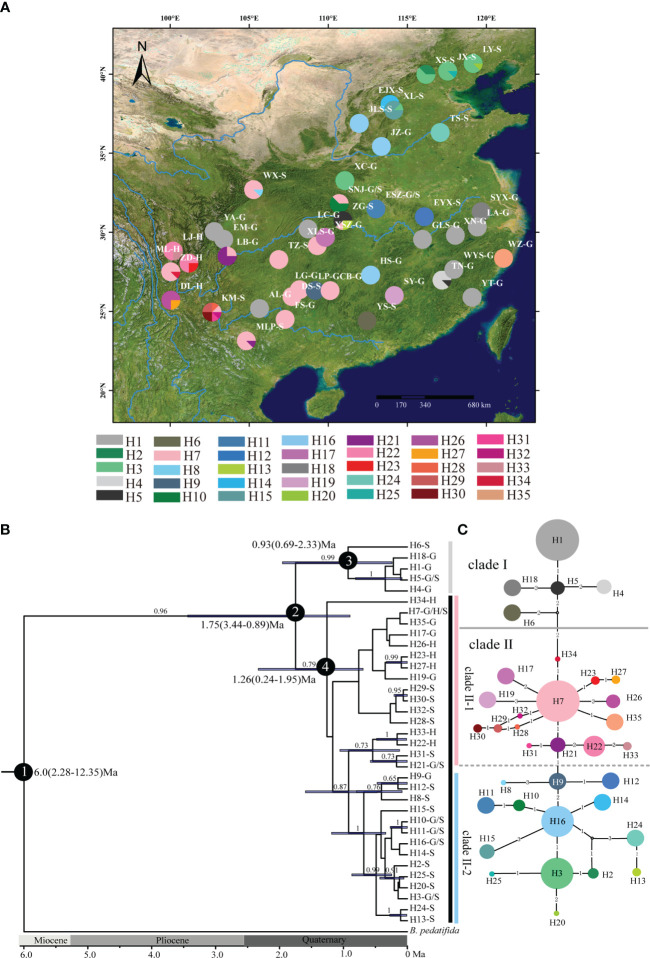
Analysis of cpDNA haplotypes of *B. grandis*. **(A)** Geographic distribution of 35 cpDNA haplotypes detected. The colored pie charts represent the frequency of haplotypes at each sampling site. Haplotype colors corresponding to the charts are displayed in the bottom panel. **(B)** BEAST-derived chronograms for cpDNA haplotypes. Numbers on branches are Bayesian posterior probabilities (shows posterior probability over 0.5 on branches). The blue bar length represents the 95% HPD of the species divergence time. **(C)** Haplotypes of the cp DNA network. The size of the circles represents the frequency of haplotypes in populations. Capital G, S, and H after population code with “-” in **(A)** and after haplotypes with “-” in **(B)** mean the subspecies identification results based on morphological classification, *B. grandis* subsp. *grandis*, *B. grandis* subsp. *sinensis*, and *B. grandis* subsp. *holostyla*, respectively.

The lengths of the *ndh*F-*rpl*32, *atp*I-*atp*H, and *ndh*A intron sequences ranged from 708 to 875, 747 to 847, and 716 to 1072 bp, respectively. The combined length of the three aligned chloroplast fragments was 2,164 bp. Based on the three sequences, 35 haplotypes representing 46 polymorphic sites, including three singleton variable sites and 43 parsimony informative sites, were identified from 352 samples (**Table 1** and **Figure 1**). Haplotype diversity (*H*_d_) and nucleotide diversity (*π*) were 0.894 and 2.9 × 10^−3^, respectively (**Table 2**). The *H*_d_ and *π* within populations were 0–0.929 and 0–3.56 × 10^−3^, respectively (**Table 1**). Total gene diversity (*H*_T_) and within-population gene diversity (*H*_S_) were estimated to be 0.910 and 0.138, respectively (**Table 2**). Only 15 populations represented diversity, and only two populations (ZG and KM) had more than two haplotypes (**Table 1**). The KM (*H*_d_ = 0.929, *π* = 1.02 × 10^−3^) and ZG (*H*_d_ = 0.607, *π* = 3.56 × 10^−3^) populations had the highest haplotype diversity and the highest nucleotide diversity, respectively (**Table 1**). Only 7 of the 35 haplotypes (H1, H3, H5, H7, H16, H21, and H22) were shared in two or more populations, while each of the remaining 28 haplotypes (80%) occurred only in a single population (**Table 1** and **Figure 1A**). H7 was the most common haplotype (found in 13 populations, with a frequency of 21.6%), followed by H1 (found in 9 populations, with a frequency of 20.5%), and these haplotypes were mainly distributed south of the Yunnan-Guizhou Plateau and Yangtze River, respectively (**Table 1**).

**Table 2:** Genetic diversity of the *B. grandis* at clade level based on cpDNA.

Clade	*H*_d_	*π* × 10^-3^	*H*_T_ (se)	*H*_S_ (se)	*G*_ST_ (se)	*N*_ST_ (se)
All data	0.894	2.9	0.910 (0.0255)	0.138 (0.0336)	0.848 (0.0359)	0.917 (0.0309)*
Clade I	0.476	0.82	0.537 (0.1638)	0.019 (0.0192)	0.964 (0.0318)	0.979 (0.0205)(NS)
Clade II	0.877	1.99	0.897 (0.0387)	0.184 (0.0439)	0.795 (0.0483)	0.851 (0.0449)*
Clade II-1	0.683	0.88	0.665 (0.1176)	0.165 (0.059)	0.751 (0.074)	0.778 (0.072)(NS)
Clade II-2	0.868	1.44	0.936 (0.033)	0.096 (0.040)	0.897 (0.044)	0.953 (0.023)*

*H*_d_, haplotype diversity; π, nucleotide diversity; *H*_T_, total gene diversity; *H*_S_, within-population gene diversity; NS, not significant, * means p < 0.05.

Based on the BEAST tree, the haplotypes H1, H4, H5, H6, and H18 were grouped into clade I, while the remaining haplotypes (H2, H3, H7–H17, and H19–H35) were grouped into clade II (**Figure 1B**). The genetic diversity of the populations in clade I (*H*_d_ = 0.476, *π* = 0.82 × 10^−3^, *H*_T_ = 0.537, *H*_S_ = 0.019) was lower than that of the populations in clade II (*H*_d_ = 0.877, *π* = 1.99 × 10^−3^, *H*_T_ = 0.897, *H*_S_ = 0.184) (**Table 2**). BAPS results suggested the existence of three geographical clusters, namely, clade I (the same as in the BEAST tree), clade II-1 (H7, H17, H19, H21–H23, H26–H35), and clade II-2 (H2, H3, H8–H16, H20, H24, H25) (**Figures 1B, C** and **Supplementary Figure S1**). Clade II-1 was mainly located in the southern part of its distribution range, while clade II-2 was mainly found in the northern part. The genetic diversity of the populations in clade II-1 (*H*_d_ = 0.683, *π* = 0.88 × 10^−3^, *H*_T_ = 0.665, *H*_S_ = 0.165), and clade II-2 (*H*_d_ = 0.868, *π* = 1.44 × 10^−3^, *H*_T_ = 0.936, *H*_S_ = 0.096) were calculated (**Table 2**).

Non-hierarchical AMOVA revealed high genetic differentiation at the species level (*F*_ST_ = 0.8352, *P* < 0.001), with 83.52% of the total variation found among populations, and only 16.48% within populations (**Table 3**). Genetic differentiation in clade I (*F*_ST_ = 0.984) was greater than that in clade II (*F*_ST_ = 0.774). However, hierarchical AMOVA indicated that 39.96% of this differentiation was distributed between clade I and clade II, 48.13% was explained by variation among populations within clades, and only 11.90% was found within populations (**Table 3**). In terms of the three geographic groups, hierarchical AMOVA indicated that 70.62% of this differentiation was distributed between three geographic groups (clade I, clade II-1, and clade II-2), 25.86% was explained by variation among populations within clades, and only 3.52% was found within populations (**Table 3**).

**Table 3:** Analyses of molecular variance (AMOVA) based on cpDNA for populations of *B. grandis*.

Source of variation	d.f.	Sum of squares	Variance components	Percentage of variation(%)	Fixation index
All					
Among populations	43	2180.756	6.187	83.52	*F*_ST_=0.835
Within populations	308	376	1.221	16.48	
Total	351	2556.756	7.408		
Clade I					
Among populations	12	234.916	2.517	98.44	*F*_ST_=0.984
Within populations	88	3.5	0.040	1.56	
Clade II					
Among populations	31	1375.965	5.458	77.39	*F*_ST_=0.774
Within populations	219	349.167	1.594	22.61	
Two clade (Clade I + Clade II)					
Among groups	1	593.208	3.856	39.96	*F*_CT_=0.400
Among populations within groups	43	1610.881	4.644	48.13	*F*_SC_=0.801
Within populations	307	352.667	1.149	11.90	*F*_ST_=0.881
Three geographic regions (Clade I, Clade II-1, and CladeII-1)					
Among groups	2	632.875	2.650	70.620	*F*_CT_=0.706
Among populations within groups	45	325.781	0.970	25.860	*F*_SC_=0.880
Within populations	305	40.25	0.132	3.520	*F*_ST_=0.965

Fixation index values were significant at all levels. d.f. means the degree of freedom.

A permutation test (**Table 2**) showed that there was significant phylogeographic structure at the species level (*G*_ST_/*N*_ST_ = 0.848/0.917, *P* < 0.05) and in clade II (*G*_ST_/*N*_ST_ = 0.797/0.851, *P* < 0.05), but not in clade I (*G*_ST_/*N*_ST_ = 0.964/0.979, *P* > 0.05). Clade II-1 (*G*_ST_/*N*_ST_ = 0.751/0.778, *P* > 0.05) exhibited no significant phylogeographic structure, but clade II-2 (*G*_ST_/*N*_ST_ = 0.897/0.953, *P* < 0.05) did (**Table 2**). Mantel test results indicated that there was a significant correlation between genetic and geographical distance matrices (*R* ^2^ = 0.022, *P* = 0.007, **Supplementary Figure S2**), indicating an isolation-by-distance effect among *B. grandi*s populations. The findings of neutrality tests (Tajima’s *D* = −0.501, *P* = 0.348; Fu’s *Fs* = 4.639, *P* = 0.850) indicated that this species had not recently undergone demographic expansion at the species level. Populations in geographic regions clade I (Tajima’s *D* = −0.294, *P* = 0.365; Fu’s *Fs* = 2.387, *P* = 0.892) and clade II-2 (Tajima’s *D* = −0.776, *P* = 0.231; Fu’s *Fs* = 2.002, *P* = 0.285) had also not undergone demographic expansion. The investigation of mismatch distributions revealed multimodal distributions that are in line with stable population size (**Supplementary Figure S3**). While, clade II-1 (Tajima’s *D* = −1.408, *P* = 0.052; Fu’s *Fs* = −5.515, *P* = 0.036) unfolded demographic expansion in neutrality tests inconsistent with the investigation of mismatch distributions that revealed multimodal distributions (**Supplementary Figure S3**).

### 3.2 Phylogenetic analysis and divergence time

The BEAST tree supported the monophyly of *B. grandis* with a posterior probability (PP) of 0.96. Thirty-five haplotypes were clustered into two clades (clade I PP = 0.99; clade II PP = 0.79) (**Figure 1B**). *Begonia grandis* and *B. pedatifida* diverged c. 6.0 million years ago (Mya) with the 95% highest probability density (HPD) date ranging from 2.28 to 12.35 Mya (**Figure 1B**; node 1). Moreover, the divergence time between clade I and clade II was estimated at 1.75 Mya (95% HPD: 3.44–0.89 Mya; node 2). The lineage diversification of clades I and II was estimated at 0.93 Mya (clade I lineage 95% HPD: 0.69–2.33 Mya; node 3) and 1.26 Mya (clade II lineage 95% HPD: 0.24–1.95 Mya; node 4), respectively. The network analysis resolved two haplotype clades, which was consistent with the haplotype structure of the BEAST tree (**Figure 1C**).

### 3.3 Species distribution model and dispersal corridors

**Figure 2:**
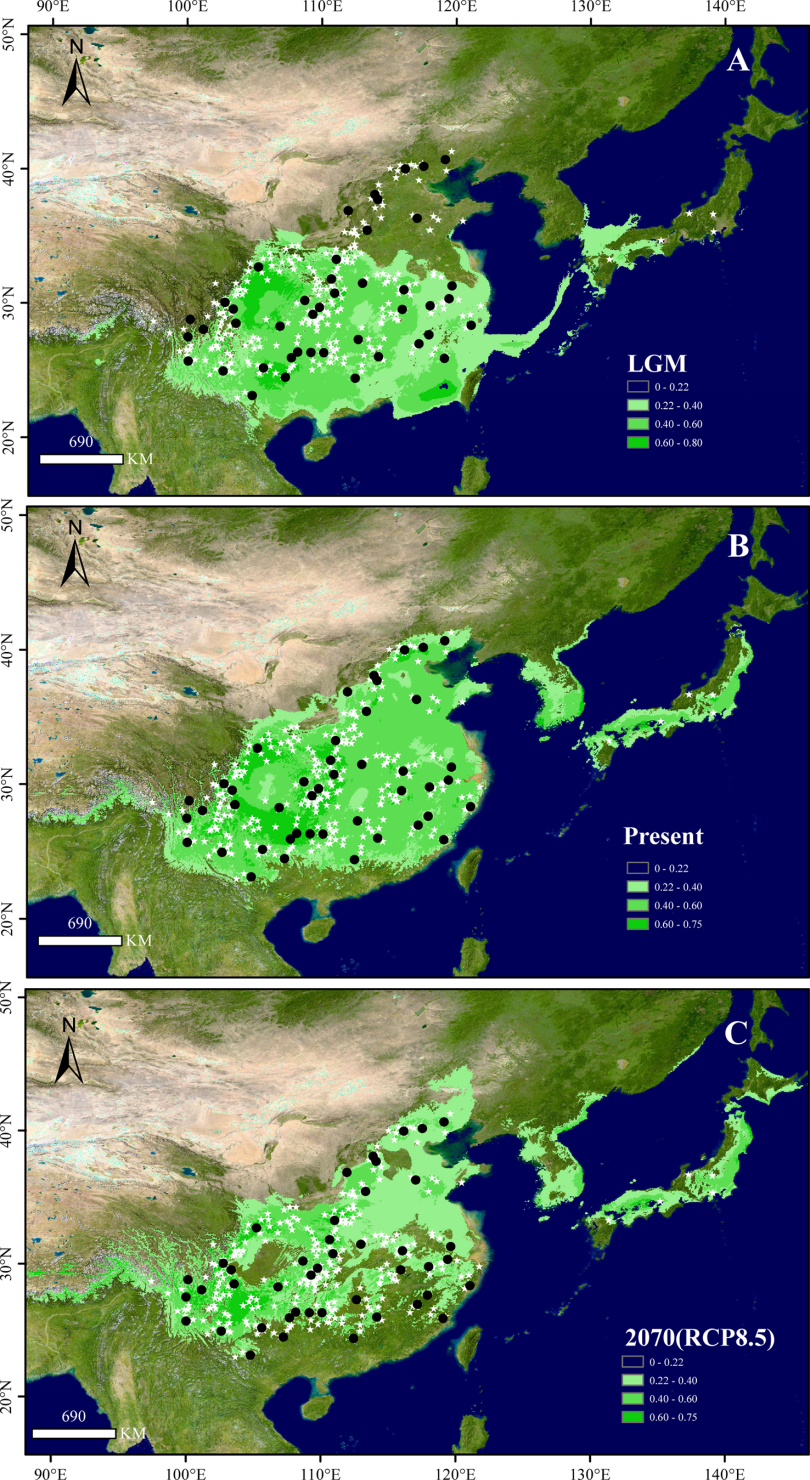
Potential distribution of *B. grandis* at the **(A)** last glacial maximum (LGM), **(B)** present, and **(C)** 2070 under the RCP8.5 scenario. Black dots show the sampling sites used in this study, and white stars show the occurrence sites obtained from herbarium records. The colors in **(A-C)**, from light to dark green, represent the fitness zone from low to high. The maximum training sensitivity plus specificity threshold (0.220 ± 0.024) was used to determine the species presence threshold.

The maxent model showed high predictive power with a high AUC value (0.955 ± 0.002). The present distributional predictions of the species were consistent with its extant natural distribution (**Figure 2**). Species distribution maps were generated with the value of the maximum training sensitivity plus specificity logistic (0.220 ± 0.024) serving as the species absence/presence threshold. The comparison of the distribution of the three periods (LGM, present, and future) indicated that (1) after the LGM, *B. grandis* underwent northward migration, and the total distribution area of the present was greater than that of the LGM (*N*_a_ = 1.12, *N*_e_ = 37.17%); (2) from the present to the future (2070), the potential range of *B. grandis* will experience northward migration, with the loss of distribution area occurring mainly in parts of central and southern China (*N*_a_ = 1.24, *N*_e_ = 32.37%) (**Supplementary Figure S4**). Precipitation of the warmest quarter (bio18) explained more than half of the variation (51.53% ± 0.92), followed by annual mean temperature (bio1) (41.0% ± 1.30) and temperature seasonality (bio4) (3.98 ± 0.64) in the identification of the areas of *B. grandis* occurrence.

**Figure 3:**
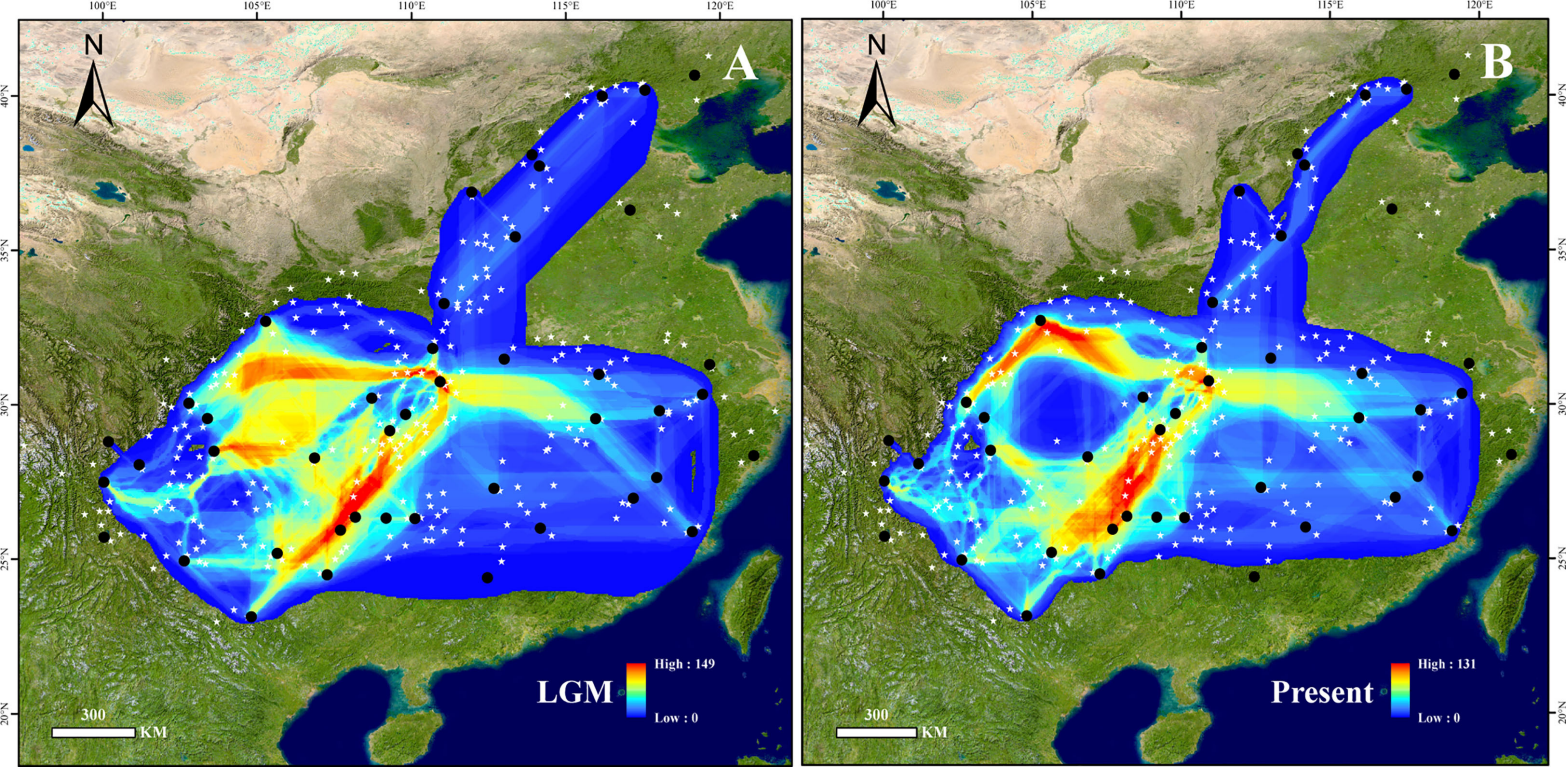
Potential dispersal corridors of *B. grandis* at the **(A)** last glacial maximum (LGM) and **(B)** present. Black dots show the sampling sites used in this study, and white stars show the occurrence sites obtained from herbarium records. The colors in **(A, B)**, from blue to red, represent the potential of species dispersal corridors from low to high.

The supposed dispersal corridors of the LGM and the present were visualized based on SDM results (**Figure 3**). The dispersal routes across periods revealed that dispersal generally centered on the Yunnan-Guizhou plateaus and the mountains surrounding the Sichuan Basin. The Wuling Mountains were crucial in the north-south migration of this species, while the northern end of the Sichuan Basin, the Wushan Mountains, and the Daba Mountains served as routes for east-west migration. The Yangtze River valley east of the Wushan Mountains served as a vital link between the eastern and western populations.

## 4 Discussion

### 4.1 Genetic diversity and genetic differentiation

In this study, we found that *B. grandis* has high genetic diversity (*H*_d_ = 0.894, *π* = 2.9 × 10^−3^, *H*_T_ = 0.910), which is very similar to that found for *B. heracleifolia* Schltdl. & Cham. (*H*_T_ = 0.937), another widespread species of *Begonia* (Twyford et al., 2013). *Begonia grandis* is extensively distributed in China, from the northern edge of the tropics, across vast subtropical regions, to the temperate region of the north of the country. Such high environmental heterogeneity and habitat fragmentation may lead to geographical isolation among populations, thereby increasing the probability of drift and mutation and, consequently, increasing genetic diversity across populations. This result was consistent with a previously stated view that widespread species tend to have higher genetic diversity than narrowly distributed ones (Hamrick and Godt, 1996).

We found *B. grandis* displayed high total gene diversity (*H*_T_ = 0.910), low genetic diversity within populations (*H*_S_ = 0.138), and variation primarily among populations (83.52%), suggesting that the genetic diversity of this species was mainly due to interpopulation differences. Notably, we detected significant genetic differentiation (*F*_ST_ = 0.835) and phylogeographic structure (*G*_ST_ < *N*_ST_, *P* < 0.05) in this species. *Begonia grandis* inhabits stone walls, slopes, waterfalls, stone crevices, and caves along gullies. It is also highly dependent on a specific microenvironment and has requirements for water, relative humidity, light, and temperature within specific ranges. Accordingly, *B. grandis* is usually fragmented and sporadic in distribution, and most of its populations are small (Li et al., 2014). Temperate forests in the Sino-Japanese Floristic Region were more considerably fragmented during the LGM than today, resulting in even more pronounced habitat discontinuities (Metcalfe et al., 2000; Cárdenas et al., 2011; Qiu et al., 2011). Even though begonias have tiny seeds (1–2 mm long), wind dispersal does not seem to be an effective dispersal mechanism in sheltered forests (Hughes and Hollingsworth, 2008). Geographic isolation and limited seed dispersal may result in minimal gene flow among populations. Small and isolated populations will experience significant genetic drift, resulting in a loss of genetic diversity within populations and an enhancement of genetic differentiation between populations (Quinn and Harrison, 1988). Genetic drift and local adaptations may have contributed to the huge diversity of leaf morphologies in *B. grandis.* Previous genetic studies of *Begonia* have also shown limited dispersal, gene flow, and strong genetic differentiation between populations in discontinuous habitats (Matolweni et al., 2000; Twyford et al., 2013; Nakamura et al., 2014). In addition, *B. grandis* often propagates *via* asexual bulbils, and large natural populations of this plant may be primarily clonal (Nakata et al., 2012), which may also explain the low average within-population genetic diversity.

### 4.2 Potential glacial refugia and dispersal corridors

Glacial refugia are geographic regions that retain habitats suitable for the persistence of a species during glacial periods, especially in the LGM, and represent locations of postglacial recolonization (Comes and Kadereit, 1998). The mountains in subtropical China became some of the most important refugia during the Quaternary glacial period (Qiu et al., 2011). Populations in refugia frequently exhibit higher levels of genetic diversity (Comes and Kadereit, 1998). However, genetic diversity can also be promoted by the geographical overlap of different populations and the hybridization or introgression between closely related species (Petit et al., 2003). Species distribution model analysis is not affected by interspecies interaction and dispersal ability. Therefore, SDMs can more objectively and precisely simulate the potential distribution of species in different periods, providing a valid basis and vital reference for the identification of refugia (Elith and Leathwick, 2009).

The notable genetic differentiation and phylogeographic structure of *B. grandis* suggested the existence of multiple glacial refugia. SDM analysis showed that the Yunnan-Guizhou Plateau, the Three Gorges region, and the Daba Mountains represented stable habitats suitable for the persistence of *B. grandis* since the LGM (**Table 1** and **Figure 2**). Meanwhile, the KM population of the Yunnan-Guizhou Plateau exhibited the highest haplotype diversity among all the populations, with six haplotypes (H7, H28, H29, H30, H31, and H32; *H*_d_ = 0.929) as well as high nucleotide diversity (*π* = 1.02 × 10^−3^). The ZG population in the Three Gorges region had the highest nucleotide diversity (*π* = 3.56 × 10^−3^) and also a very high haplotype diversity (*H*_d_ = 0.607). Populations (SNJ and WX) in the Daba Mountains also had high genetic diversity (SNJ: *π* = 1.37 × 10^−3^, *H*_d_ = 0.54; WX: *π* = 0.64 × 10^−3^, *H*_d_ = 0.25). We speculate that the Yunnan-Guizhou Plateau, the Three Gorges region, and the Daba Mountains were glacial refugia and dispersal corridors for *B. grandis* based on the long-term stability of the habitats and the high genetic diversity of the local populations (**Table 1**, **Figures 1A**, **2**, **3**). Although the populations (XS, XL, and JX) in north China also displayed high genetic diversity, they had no suitable habitats during the LGM (XS: *N*_LGM_ = 0.05; XL: *N*_LGM_ = 0.02; JX: *N*_LGM_ = 0.01), and their haplotypes were near the end of the haplotype network rather than being ancestral. This suggested that these populations were not derived from refugia but were a result of northward migration after the glacial period. The eastern populations may have migrated through the Daba Mountains, the Wushan Mountains, and the Yangtze River valley, while the northern populations may have migrated through the Taihang Mountain Range, although the dispersal corridors analysis only weakly supported this possibility. Notably, because *B. grandis* has significant ornamental value, some eastern populations may have been introduced as ornamental plants from Southwest China, such as population YT found in hills beside temples, and populations XN, LA, and WYS that are found in nature reserves or forest parks. Having asexual bulbils and underground tubers, which become dormant in winter, *B. grandis* is well adapted to low temperatures, which may explain why its habitat has expanded northward since the LGM.

### 4.3 Subspecies classification

The results of the phylogenetic and haplotype network analysis do not support subspecies classification of the FRPS and FOC based on morphological characteristics (Ku, 1999; Gu et al., 2007). There were three possible reasons for this discrepancy. First, we did not use enough chloroplast markers in our analysis, and thus our results cannot accurately reflect the current subspecies classification. Secondly, the classification based on morphology was unreasonable or inaccurate because the key morphological traits were continuous. Thirdly, the subspecific classification in both FRPS and FOC may not have reached the subspecies division level (Ku, 1999; Gu et al., 2007). Based on the comprehensive results of the current and previous studies, we think that the latter two reasons are more likely (Li et al., 2021). For example, one population may have individuals with red, purple, and green abaxial leaves and may have large variations in leaf and leaf edge shapes. Additionally, the degree of filament connation does not differ markedly between populations. Although *B. grandis* subsp. *holostyla* Irmsch., distributed in northwestern Yunnan province, is styles-free and has an unbranched stigma, unlike the populations in other places of this province, it is not monophyletic based on molecular marker analysis. This group may be a transitional type and cannot, therefore, be treated as a subspecies. Our results suggested that *B. grandis* should not be divided into subspecies as done by FRPS and FOC, and more morphological and genomic data must be combined to support subspecies treatment in future work.

### 4.4 Suggestions for resource conservation and utilization

*Begonia grandis* has important ornamental value and is often cultivated as a landscape plant in scenic or tourist sites in China. It is also an important traditional medicine. Effective protection of the wild sources of *B. grandis* is a basic requirement for the sustainable use of this species. Based on the pattern of genetic diversity distribution and the results of SDM, populations with high genetic diversity, unique haplotypes, stable habitats, special traits, or extreme distribution should be protected *in situ* or conserved *ex situ* as a priority. Specifically, the populations in southwest regions with potential as refugia, such as the Yunnan-Guizhou Plateau, the Three Gorges region, and the Daba Mountains, have high genetic diversity, and their habitats are stable. The populations in Lingyuan City of Liaoning Province and Chayu County of Tibet Province are in extreme distribution points and have the highest cold resistance. Attention should also focus more on the conservation and utilization of some populations with special traits, such as those with stable, white-spotted leaves in Yongshun county of Hunan province. Meanwhile, as the populations of southern China are the most vulnerable to climate change, they should be continuously monitored.

## 5 Conclusions and perspectives

*Begonia grandis* is more tolerant to low temperatures than any other species of *Begonia*. The high haplotype diversity and total gene diversity of *B. grandis* may indicate that its adaptation to low temperatures allowed it to survive in small but ecologically suitable and isolated habitats throughout the Pleistocene. Thus, its niche has undergone a broad expansion, especially after the glacial periods. The asexual reproductive bulbils in the leaf axils were an important feature in the short-distance dispersal of this species. Given their significant ornamental value, some populations of *B. grandis* located near temples and scenic sites in eastern China may have been introduced from southwest China. The characteristics of *B. grandis* mentioned above increase its competitiveness and may be an important reason for its wide distribution. Like other begonias, the seeds, pollens, and bulbils of *B. grandis* have limited dispersal ability in relatively closed forests. Moreover, *B. grandis* strongly depends on a specific microenvironment. This leads to fragmented geographical distribution patterns that limit gene flow, which may be an important reason for the significant genetic differentiation between populations. The population genetic structure of *B. grandis*, with high total genetic diversity, high haplotype diversity, low within-population gene diversity, and significant genetic differentiation between populations, is consistent with a macroevolutionary pattern (Hughes and Hollingsworth, 2008). Restricted gene flow and solid reproductive barriers make it possible to maintain both inter- and intra-species boundaries, which may explain the rich diversity of species in *Begonia* as well as the significant genetic differentiation at the population level (Hughes and Hollingsworth, 2008; Twyford et al., 2014; Twyford et al., 2015). *Begonia grandis* provides an excellent example for the study of the population genetics of widespread begonias. Combined with previous cases (e.g., *B. heracleifolia, B. fenicis* Merr*., and B. luzhaiensis* T. C. Ku), our study sheds light on the evolutionary mechanisms responsible for generating and maintaining species diversity in one of the world’s largest plant genera. However, the information on polymorphism based on the chloroplast markers used in our study is limited. Further genome-wide data are needed to more thoroughly reveal the population genetic structure and population history dynamics of *B. grandis*. In addition, population genetic studies of other widespread species, such as *B. longifolia, B. palmata*, *B. handelii*, and *B. fimbristipula*, are also warranted.

## Data availability statement

The datasets presented in this study can be found in online repositories. The names of the repository/repositories and accession number(s) can be found below: https://www.ncbi.nlm.nih.gov/, OQ433949-OQ433994.

## Author contributions

D-KT, CL, X-LJ, and YX designed the study; D-KT, X-JL, X-PL, CL, and YX conducted the field surveys; X-JL and YX performed the laboratory work; YX and X-LJ analyzed the data; YX, X-LJ, and D-KT prepared the manuscript; D-KT, YX, X-LJ, and W-PL revised the manuscript. All authors contributed to the article and approved the submitted version.

## Footnotes

1
https://padme.rbge.org.uk/begonia/home

2
http://www.esri.com/software/arcgis/

3
http://tree.bio.ed.ac.uk/software/tracer

4
http://tree.bio.ed.ac.uk/software/figtree/

5
https://www.cvh.ac.cn/

6
http://www.nsii.org.cn/2017/home.php

7
http://www.worldclim.org/

8
https://www.r-project.org/
