# Supplementary material for: Spatial genetic patterns and distribution dynamics of Begonia grandis (Begoniaceae), a widespread herbaceous species in China
Source: Front Plant Sci. 2023 May 10;14:1178245. doi: 10.3389/fpls.2023.1178245 (PMC10206317; doi:10.3389/fpls.2023.1178245)
Supplement: Supplementary file 1 [file DataSheet_1.doc]

Supplementary Material

# Spatial Genetic Patterns and Distribution Dynamics of *Begonia grandis* (Begoniaceae), a Widespread Herbaceous Species in China

Yan Xiao, Xing-Juan Li, Xiao-Long Jiang, Chun Li, Xiang-Peng Li, Wei-Ping Li*, Dai-Ke Tian*

*** Correspondence:** Wei-Ping Li: [lwp@hunnu.edu.cn](mailto:lwp@hunnu.edu.cn); Dai-Ke Tian: [dktian@cemps.ac.cn](mailto:dktian@cemps.ac.cn)

# Supplementary Figures


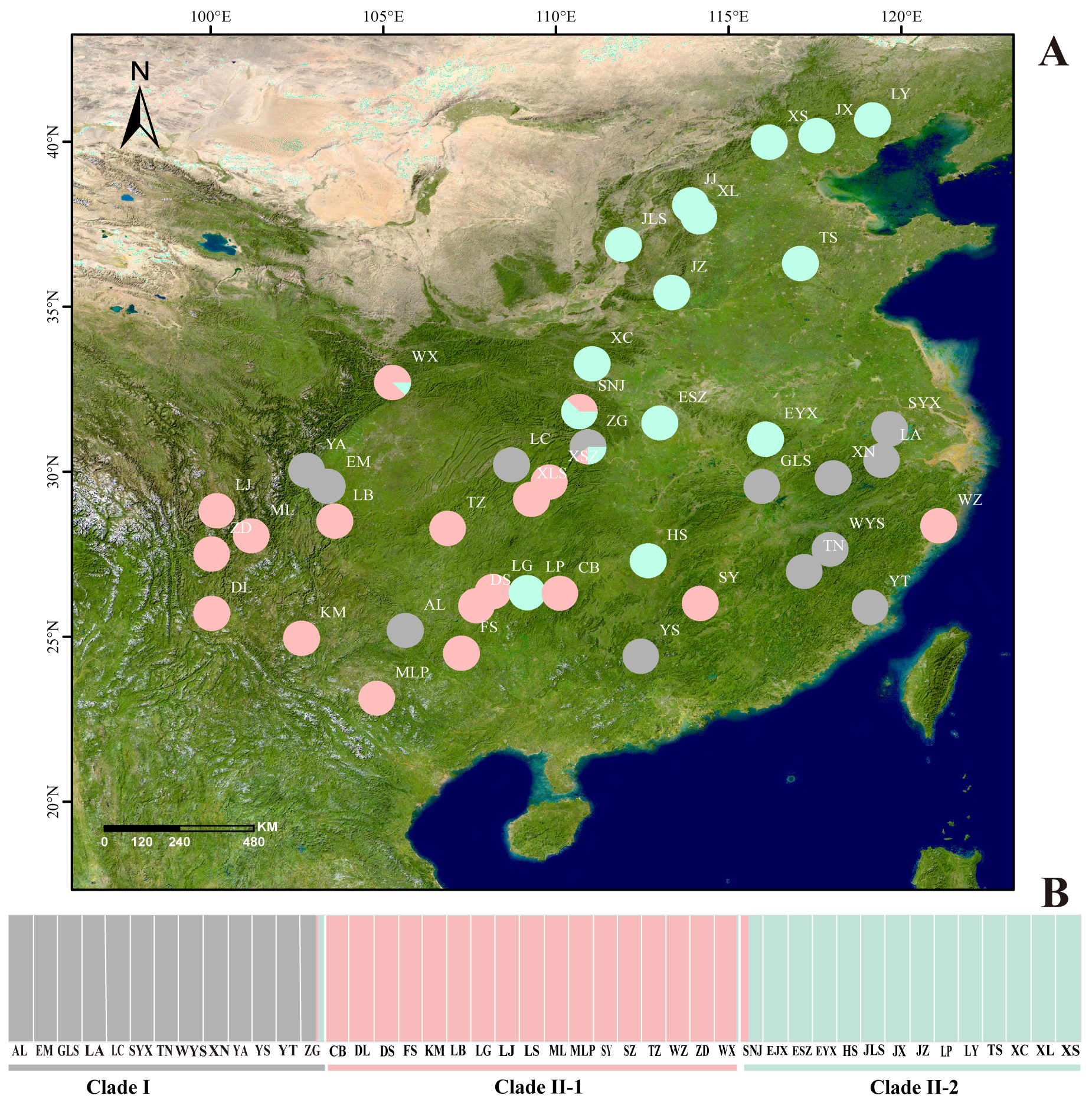


**Supplementary Figure S1.** Population genetic structure of *B. grandis*. (A) Spatial clustering of 44 populations in BAPS (*K* = 3). The gray circles represent the populations in clade I; The pink circles represent the populations in clade II-1; The green circles represent the populations in clade II-2. (B) Histogram of Bayesian admixture results in BAPS (*K* = 3). The gray histograms represent the populations in clade I; The pink histograms represent the populations in clade II-1; The green histograms represent the populations in clade II-2.

**
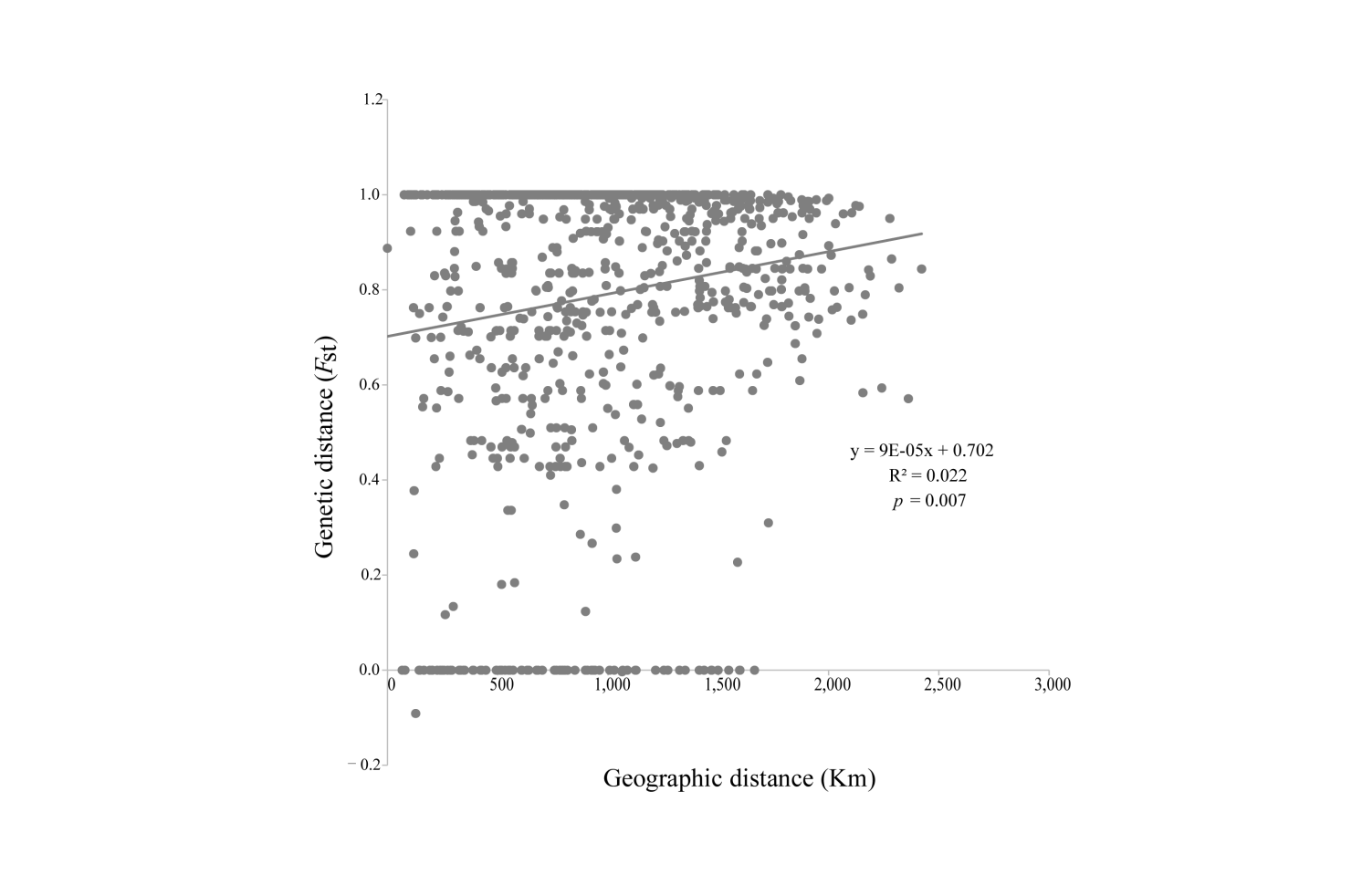
**

**Supplementary Figure S2** Relationship between geographic and genetic distances.


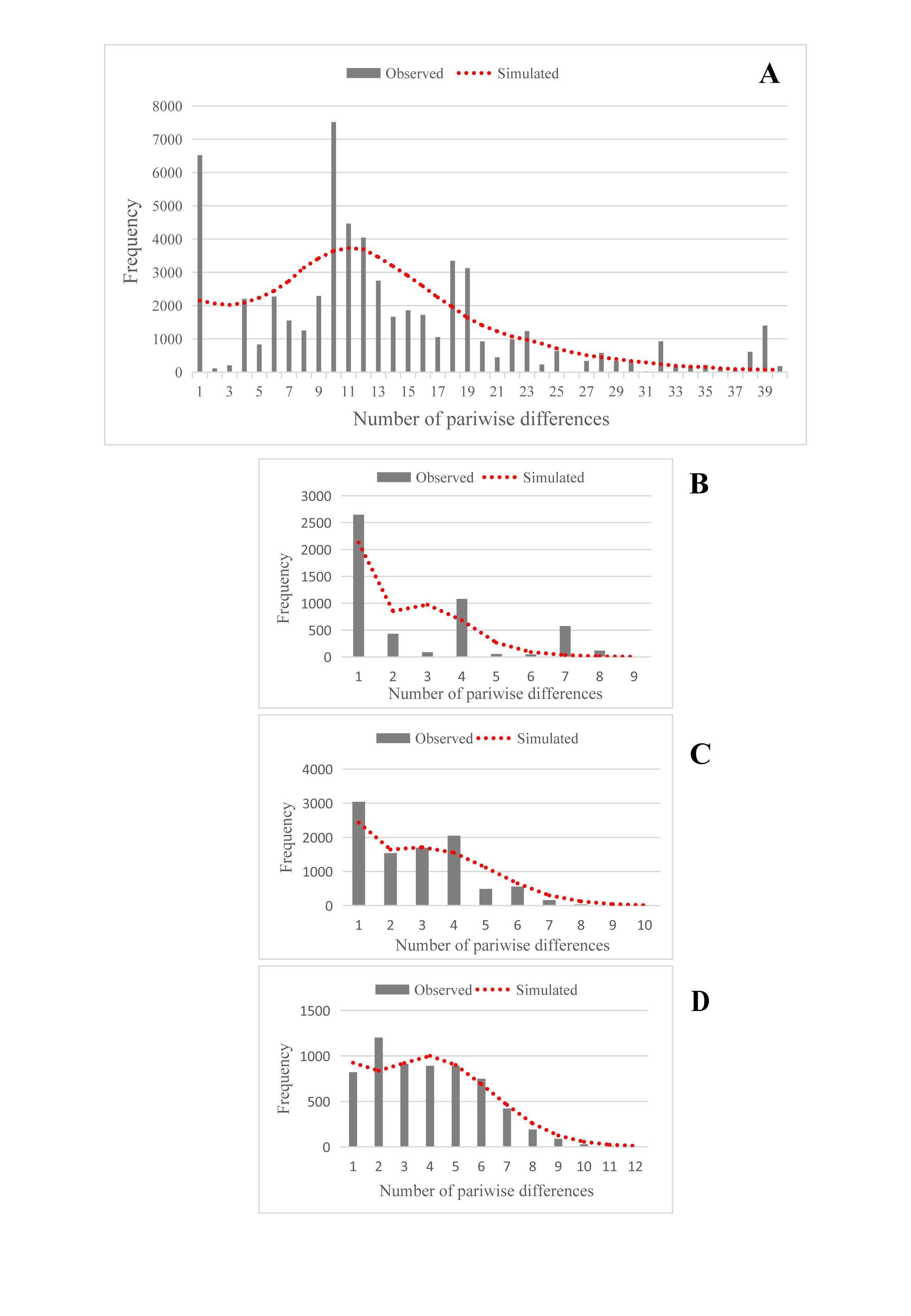


**Supplementary Figure S3** Mismatch distribution for populations of *B. grandis* at (A) the species level, (B) clade I, (C) clade II-1, and (D) clade II-2.


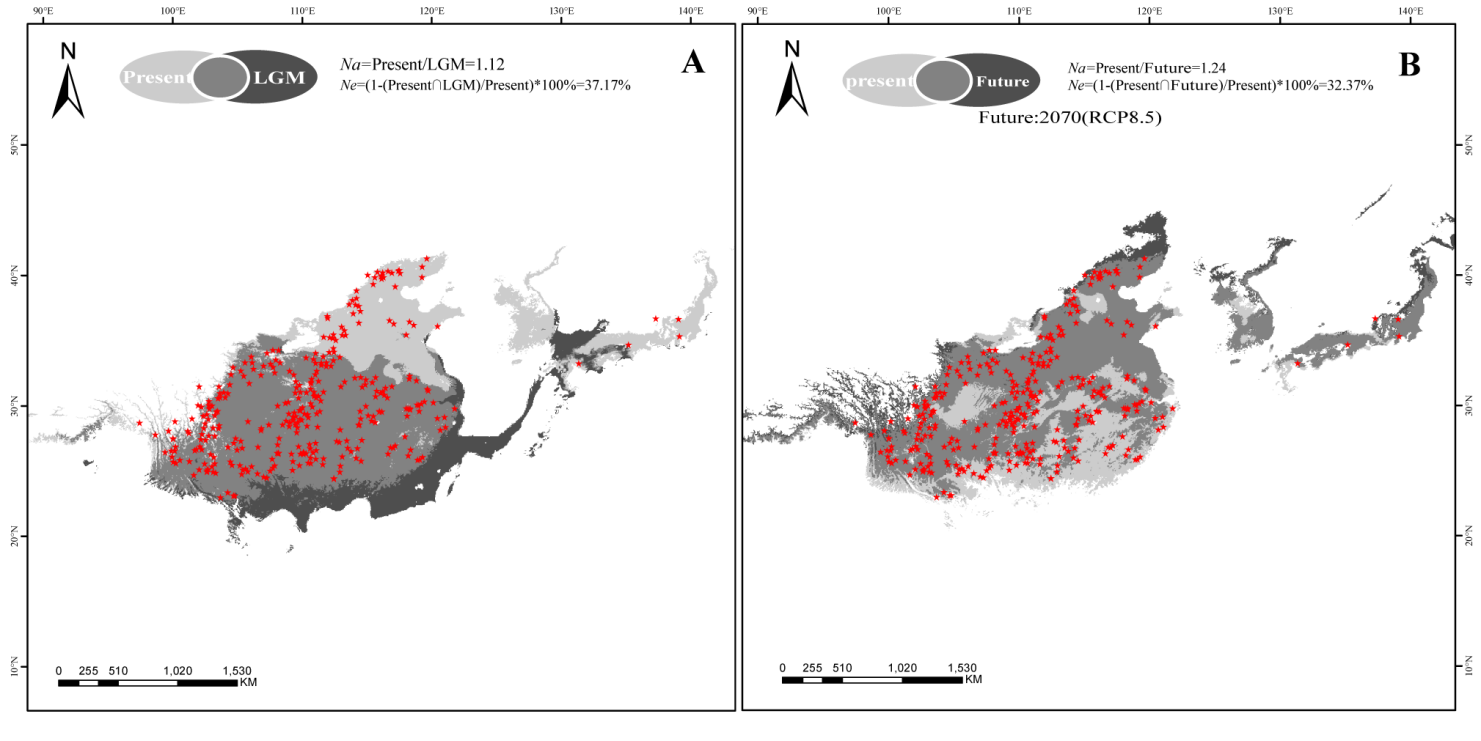


**Supplementary Figure S4** The comparison of species distribution areas between the present with LGM periods (A) and present with future (B). The maximum training sensitivity plus specificity threshold (0.220 ± 0.024) was used to determine the species presence threshold. Gray regions represent where the present and LGM/future periods overlap in distribution; light and dark gray regions represent where the species was only distributed during the present and LGM/future periods, respectively. The red stars represent the points used to simulate the species distribution. *N*_a_ and *N*_e_ represent the habitat distribution area ratio and extent of species range expansion, respectively.

**Supplementary Tables**

***Supplementary Table S1*** *cpDNA used in population genetic structure scan of B. grandis.*

| Primer | | Sequences (5'~3') | Reference |
| --- | --- | --- | --- |
| *ndh*F-*rpl*32 | *ndh*F*Beg*-F | TGGATGTGAAAGACATATTTTGCT | Thomas, 2010 |
|  | *rpl*32*Beg*-R | TTTGAAAAGGGTCAGTTAATAACAA |  |
| *atp*I-*atp*H | *atp*I | TATTTACAAGYGGTATTCAAGCT | Shaw et al., 2007 |
|  | *atp*H | CCAAYCCAGCAGCAATAAC |  |
| *ndh*A intron | *ndh*A x1 | GCYCAATCWATTAGTTATGAAATACC | Shaw et al., 2007 |
|  | *ndh*A x2 | GGTTGACGCCAMARATTCCA |  |

**Supplementary Table S2** Nineteen bioclimatic variables.

| **BIO1 = Annual Mean Temperature** |
| --- |
| **BIO2 = Mean Diurnal Range (Mean of monthly (max temp**–**min temp))** |
| **BIO3 = Isothermality (BIO2/BIO7) (× 100)** |
| **BIO4 = Temperature Seasonality (standard deviation × 100)** |
| **BIO5 = Max Temperature of Warmest Month** |
| BIO6 = Min Temperature of Coldest Month |
| BIO7 = Temperature Annual Range (BIO5-BIO6) |
| **BIO8 = Mean Temperature of Wettest Quarter** |
| BIO9 = Mean Temperature of Driest Quarter |
| BIO10 = Mean Temperature of Warmest Quarter |
| BIO11 = Mean Temperature of Coldest Quarter |
| **BIO12 = Annual Precipitation** |
| BIO13 = Precipitation of Wettest Month |
| BIO14 = Precipitation of Driest Month |
| **BIO15 = Precipitation Seasonality (Coefficient of Variation)** |
| BIO16 = Precipitation of Wettest Quarter |
| BIO17 = Precipitation of Driest Quarter |
| **BIO18 = Precipitation of Warmest Quarter** |
| BIO19 = Precipitation of Coldest Quarter |

Note: The bold means used to analyze after reducing the subsets of variables with high correlations.
